# Biomechanics of a collum-fixated short stem in total hip arthroplasty

**DOI:** 10.1016/j.jor.2024.02.027

**Published:** 2024-02-21

**Authors:** Anders Tjønneland, Poul Torben Nielsen, Thomas Jakobsen

**Affiliations:** Interdisciplinary Orthopaedics, Department of Orthopaedic Surgery, Aalborg University Hospital, Hobrovej 18-22, 9100, Aalborg, Denmark

**Keywords:** Total hip arthroplasty, Hip biomechanics, Short stem, Femoral offset, Global offset, Neck shaft angle

## Abstract

**Background:**

Biomechanical reconstruction of the hip significantly impacts the clinical outcome and implant survival. Our knowledge is limited of the ability of neck-stabilised prostheses to restore hip biomechanics. We hypothesised that hip biomechanics, specifically leg length and global offset (GO), may be restored to an acceptable range using the Primoris™ stem.

**Methods and material:**

In this retrospective study, we analysed 152 patients who underwent total hip replacement (THA) using the short collum-fixated stem Primoris™.

The primary outcomes were hip parameters measured by x-ray following THA using the Primoris™ stem. After surgery, the biomechanical parameters used were measured at the arthroplasty and the native contralateral side of the same x-ray. The X-rays were taken one year after the patient's surgery.

1. GO.

2. Leg length discrepancy (LLD).

3. Neck shaft angle (NSA).

**Results:**

We recorded an average GO of −3.4 mm (standard deviation (SD) 7.2) and an average LLD of +3.8 mm (SD 6.4). Furthermore, we registered an average 14-degree NSA increase (SD 7.4).

**Conclusion:**

The Primoris™ neck-stabilised stem enabled hip anatomy restoration to a favourable range with respect to GO and LLD as the average difference fell within ±5 mm. However, the stem tended to be implanted in valgus.

## 1 Introduction

Total hip arthroplasty (THA) has become one of the most successful procedures in orthopaedic surgery. Therefore, surgery indications have been widened to include younger and more active patients. Scandinavian data show that a third of the patients receiving a THA are younger than 65 years.^1^ In this younger and more active group, the biomechanical forces on the hip implant are higher and the life expectancy of the patient is longer. Some of the derived challenges are polyethylene (PE) wear, osteolysis, component or periprosthetic fracture, corrosion and stress shielding. As a consequence, revision rates will inevitably rise.^2^

Short-stem implants have been developed and used intermittently since Philip Wiles’ hip replacement in 1938^3^ and the introduction of the Judet prosthesis in 1947.^4^ However, designs employing a short-stem femoral component remain widely unaccepted. Using a short stem has various likely benefits, including heightened physiologic loading of the proximal femur, which leads to bone stock preservation in the calcar region, avoids stem-induced thigh pain and renders revision THA less complicated.

According to the Joint Implant Surgery Research Foundation (JISRF) stem classification system,^3^ the Primoris™, Biomet-Zimmer, stem is classified as 2C; a neck-only stabilised stem. The stem was designed to have physiological loading on the proximal part of the femur, and has shown promising bone-preserving results in a pilot series.^5^

Biomechanical hip reconstruction significantly impacts the clinical outcome and implant survival.^6^, ^7^, ^8^ To the best of our knowledge, the ability of neck-stabilised prostheses to restore hip biomechanics remains unexplored.

The overall aim was to evaluate the effect of the collum-fixated stem Primoris™ on hip biomechanics. We hypothesised that hip biomechanics may be restored and, more specifically, that leg length difference (LLD) and global offset (GO) and would be appropriate. The primary outcomes were x-ray-measured hip parameters after THA using the Primoris™ stem.

## 2 Materials and methods

As part of a stepwise introduction, two studies were conducted.^9^ First, a cadaveric study and subsequently a clinical trial. We evaluated x-rays of nine cadaveric femurs before and after insertion of the collum-fixated stem and assessed 127 clinical x-rays from patients following surgery with the collum-fixated stem.

### 2.1 Cadaveric trial

**Fig. 1:**
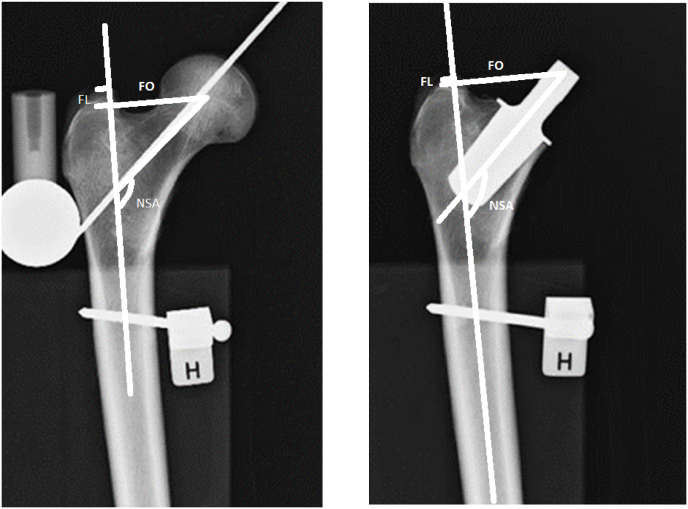
Pre- and post-operative x-rays of cadaveric femurs.

Primoris™ underwent preclinical testing in 2009 during which the implant was inserted in nine cadaveric femurs. The operative procedure was the same as described below for the clinical trial. X-rays were taken before and after surgery. The femurs were fixated on a wooden board, ensuring that the collum axis was horizontal. The x-rays thereby illustrate true femoral offset. The pre- and post-operative x-ray were taken in an identical manner. Thus, the only difference was the inserted Primoris™ stem. Therefore, the pre- and post-operative femoral x-rays are comparable (See Fig. 1).

On the cadaveric femurs, we measured femoral offset (FO), vertical femoral lengthening (FL) and neck shaft angle (NSA) before and after surgery (See Fig. 3 for further details).

### 2.2 Clinical study design

This retrospective cohort study was performed at the Farsoe Orthopaedic Clinic, Aalborg University Hospital, Aalborg, Denmark. The local ethics committee approved the study (no. N-20100054) and the study was registered with ClinicalTrials.gov (NCT01326832). All patients gave informed consent and were then enrolled following the Strengthening the Reporting of Observational Studies in Epidemiology (STROBE) guidelines on observational studies in epidemiology^10^ and in conformity with the Declaration of Helsinki.

### 2.3 Study population

From July 2011 to June 2015, a total of 1294 patients underwent a THA at the clinic, 152 of whom received the collum-fixated stem, Primoris™.

The inclusion and exclusion criteria for this group are shown in Table 1 (See Table 1).

**Table 1:** Inclusion and Exclusion criteria.

Inclusion criteria for the cohort	Exclusion criteria for the cohort
Primary hip osteoarthritis	Inflammatory joint disease (e.g., rheumatoid arthritis)
	Significant coxa vara/valga
Female patients aged 18–55 years or male patients aged 18–65 years at the time of surgery.	Rotational deformities or insufficient bone quality
	Congenital or acquired short neck segment
	Hip dysplasia
	Co-morbidity – ASA IV-V
	Pregnancy
	Body Mass Index (BMI) ≥ 35
	Neurological conditions affecting movement
	Dementia

ASA: American Society of Anesthesiologists Classification.

**Fig. 2:**
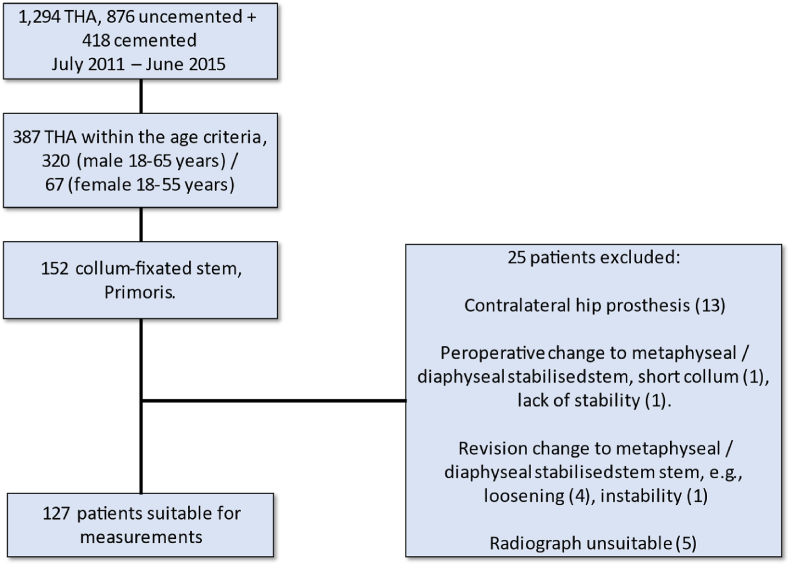
Flow diagram. THA: Total hip arthroplasty.

For this highly selected group of 152 patients, we excluded 25 patients as they were unsuitable for measurements (See Fig. 2).

**Fig. 3:**
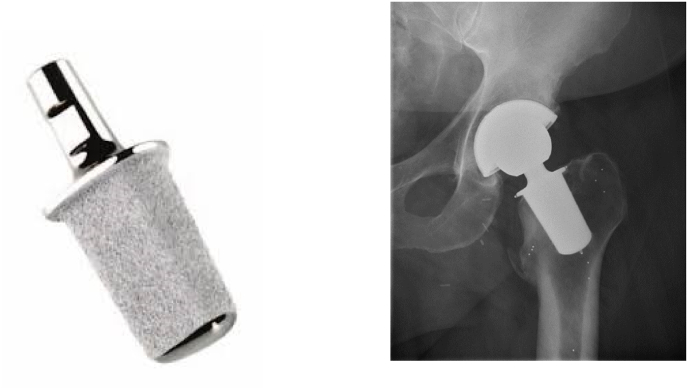
The Primoris implant. Radiographic image of the Primoris in vivo prepared for radiostereometric analysis with tantalum beads in the proximal part of the femur and three tantalum markers attached to the implant.

The remaining 127 patients had the following epidemiological data; mean age 52 years (standard deviation (SD) 8.7), a Body Mass Index of 27.7 (SD 3.8) and a female-to-male ratio of 16/111.

### 2.4 Implant

The Primoris hip implant (ZimmerBiomet, Warsaw, Indiana, US) is made of titanium and is a neck-preserving and truly collum-fixated stem. The implant is designed in a tapered and elliptical-trapezoidal shape, which fits the dimensions of the inner femoral neck. This enhances initial stability, secures osseointegration and achieves a physiological load pattern.^11^ (See Fig. 3).

The implant is coated with a 5-μm porous surface of electrochemically deposited hydroxyapatite (BoneMaster) atop a porous coating achieved by Ti-plasma spraying to optimise osseointegration.^12^ According to the JISRF stem classification system, the Primoris stem is classified as a 2C; neck-stabilised and neck-only stem.^3^

### 2.5 Surgery

Surgery was performed by two experienced orthopaedic senior surgeons adopting a posterior approach (Moore's approach). Tablets of intravenous cephalosporin and tranexamic acid were administered before and after surgery. All patients received prophylaxis of thrombosis during their hospitalisation. The implanted Primoris stem had a size ranging from 22 to 30 mm. The surgeon endeavoured to position the Primoris stem in line with the collum axis and aimed to situate the resection 25 mm proximally to the proximal part of the trochanter minor. The stem was inserted using a bone compaction technique for early fixation.^13^ The femoral heads were CoCr (36 or 32 mm). The best suitable neck length was chosen preoperatively, prioritising hip stability and leg length. Posterior stability was assessed with the hip flexed 90° and internal rotation over 45°. Anterior stability was evaluated with full hip extension and external rotation.

The cup used was Regenerex (Biomet) in the first 102 patients and Exceed (Biomet) in the last 50 patients. The median cup size was 56 (range 50–62) mm. Median stem size was 26 (range 22–30) mm, expressing stem width below the collar. The head size was 32 mm in 9% of patients and 36 mm in the remaining 91%. The neck length was −4 in 58% of patients, 0 mm in 35% and +4 mm in 8%. The bearing surfaces were E-vitamin-enriched, highly cross-linked polyethylene on CoCr.

### 2.6 Radiographic evaluation

We determined biomechanical parameters from a postoperative x-ray providing a pelvic overview. The x-ray was taken one year after surgery. The method used to measure the biomechanical parameters was adopted from previous studies.^14^^,^^15^ The radiographs were centred around the pubis and the patient was in the supine position. Furthermore, the legs of the patient were parallel, stretched and rotated 10–15° internally, lateralising the trochanter major and exposing the FO.

Radiographs were considered unsuitable provided the coccyx was more than 1 cm lateralised from the centre of the pubic symphysis or more than 3 cm above or below the superior edge of the pubis.^16^ These criteria meant that two radiographs and one radiographs were excluded.

We assessed internal rotation by measuring the width of both lesser trochanters. Radiographs were unsuitable provided the horizontal width of both lesser trochanters differed by more than 50%.^15^ This resulted in two radiographs being excluded.

**Fig. 4:**
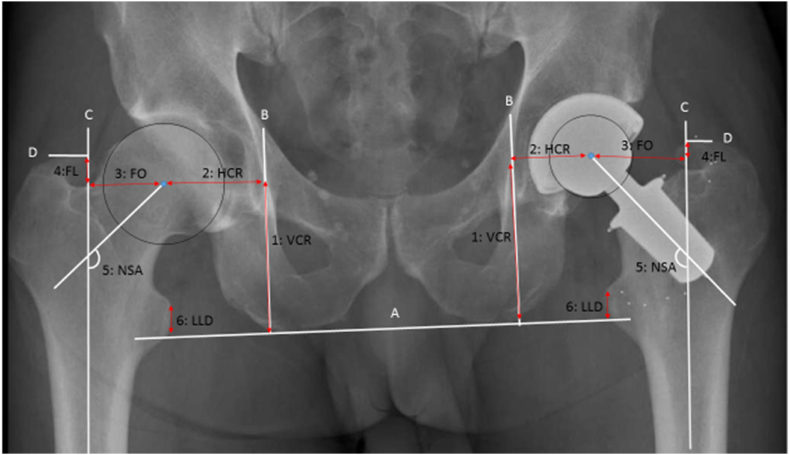
Biomechanical parameters. A = *trans*-ischial line. B = medial teardrop line, perpendicular to the *trans*-ischial line. C = femoral shaft line. D = top of trochanter major projected perpendicular to femoral shaft line. 1 = vertical hip centre of rotation (VCR), the perpendicular distance from *trans*-ischial line (A) to the level of centre of rotation. 2 = the horizontal acetabular offset (HCR), the perpendicular distance from the centre of rotation to the medial edge of the ipsilateral teardrop line (B). 3 = horizontal femoral offset (FO) is the perpendicular distance from the centre of rotation to the line bisecting the femoral long axis (C). 4 = vertical femoral lengthening (FL) was measured from two points projected perpendicular to the femoral long axis. The two points used were the centre of rotation and the top of trochanter major (D). 5 = neck shaft angle (NSA) is the angle formed by the femoral shaft line (C) and the femoral neck. The neck axis was determined from the centre of the femoral head to a line bisecting the femoral neck determined at the narrowest place of the neck. On the operated side, the NSA was the angle between a line bisecting the prosthesis and the femoral long axis. 6 = leg length discrepancy (LLD) was measured from the *trans*-ischial line (A) and the perpendicular distance to the prominent point of trochanter minor.

Biomechanical parameters (See Fig. 4).

The following parameters were measured after the operation at the arthroplasty and the native contralateral side on a single x-ray.

1. Vertical hip centre of rotation (VCR), acetabular vertical change.
2. Horizontal hip centre of rotation (HCR), acetabular horizontal offset.
3. FO
4. FL
5. NSA
6. Leg length discrepancy (LLD)

All measurements were performed digitally using TraumaCad, (Brainlab AG). A single author (AT) performed all the measurements. To assess intra-observer variation, 20 randomly selected radiographs were re-measured. Interobserver variation was assessed by having a second author (TJ) repeat the same measurements. The x-rays used to provide a postoperative overview of the pelvis were calibrated for size using a 36 or 32 mm femoral head.

### 2.7 Statistical analysis

Continuous data were presented as means with 95% confidence interval (95% CI) and SD. Furthermore, a we used the paired T-test to evaluate the biomechanics. A p value exceeding 0.05 was considered significant. Intra- and inter-observer variations were assessed by calculation of interclass correlation coefficients (ICC), where a coefficient below 0.50 was considered poor, 0.50–0.75 was moderate, whereas 0.75–0.90 was good and above 0.90 was excellent.^17^ The statistical analysis was performed using Excel 365 and StataMP version 16 (StataCorp LLC, Texas).

## 3 Results

### 3.1 Cadaveric results

On the nine cadaveric femurs, the mean change in hip parameters before and after surgery were; a +3.4 mm increase in FO, a +6.8 mm increase in vertical FL and a +8° valgus increase in NSA (Table 2).

**Table 2:** Hip parameters cadaveric and clinical.

Hip parameter				
**Cadaveric:**	Preoperative femur, mean (95% CI)	Postoperative femur, mean (95% CI)	Mean difference (SD)	
Femoral offset (mm)	42.4 (38.5–46.3)	45.8 (42.0–49.5)	+3.4 (3.5)	
Femoral lengthening (mm)	−13.4 (−16.0 to −10.7)	−6.5 (−8.9 to −4.1)	+6.8 (2.4)	
Neck shaft angle (degrees)	124 (121–127)	133 (129–137)	+8 (6.6)	
**Clinical:**	Non-operated side, mean (95% CI)	Operated side, mean (95% CI)	Mean difference (SD)	p value
Femoral offset (mm)	38.5 (37.3–39.7)	37.7 (36.4–39.0)	−0.8 (6.1)	0.34
Femoral lengthening (mm)	−6.4 (−7.2 to −5.6)	+0.4 (−0.6 to 1.4)	+6.8 (4.8)	<0.001
Leg length (mm)	6 (4.8–7.2)	2.2 (1.1–3.4)	+3.8 (6.4)	<0.001
Neck shaft angle, NSA (degrees)	130 (129–131)	144 (142–145)	+14 (7.4)	<0.001
Global offset, FO + HCR (mm).	77 (75.5–78.6)	73.7 (72.1–75.2)	−3.4 (7.2)	0.002
Vertical pelvic centre of rotation, VCR (mm)	62.8 (61.7–64.0)	66.2 (64.9–67.4)	+3.5 (5.6)	<0.001
Horizontal pelvic centre of rotation, HCR (mm)	38,6 (37.9–39.3)	36.0 (35.4–36.6)	−2.6 (3.7)	<0.001

CI: confidence interval. SD: standard deviation.

### 3.2 Clinical results

The main findings of this study were an average GO of −3.4 mm and an average LLD of 3.8 mm, i.e. both means were within the −5 to 5 mm threshold values. In this study, 41% (52/127) of the GO was lower than −5 mm, 45% (57/127) was within the range of −5 to 5 mm and 14% (18/127) exceeded 5 mm. The LLD was distributed as follows; 8% (10/127) under −5 mm, 51% (65/127) in the −5 to 5 mm range and 41% (52/127) exceeded 5 mm.

Furthermore, in comparison with the contralateral side, the operated hips had an average 3.5 mm VCR increase (p < 0.001) and a mean 2.6 mm HCR decrease (p < 0.001); moving the pelvic centre of rotation (COR) proximally and medially.

**Fig. 5:**
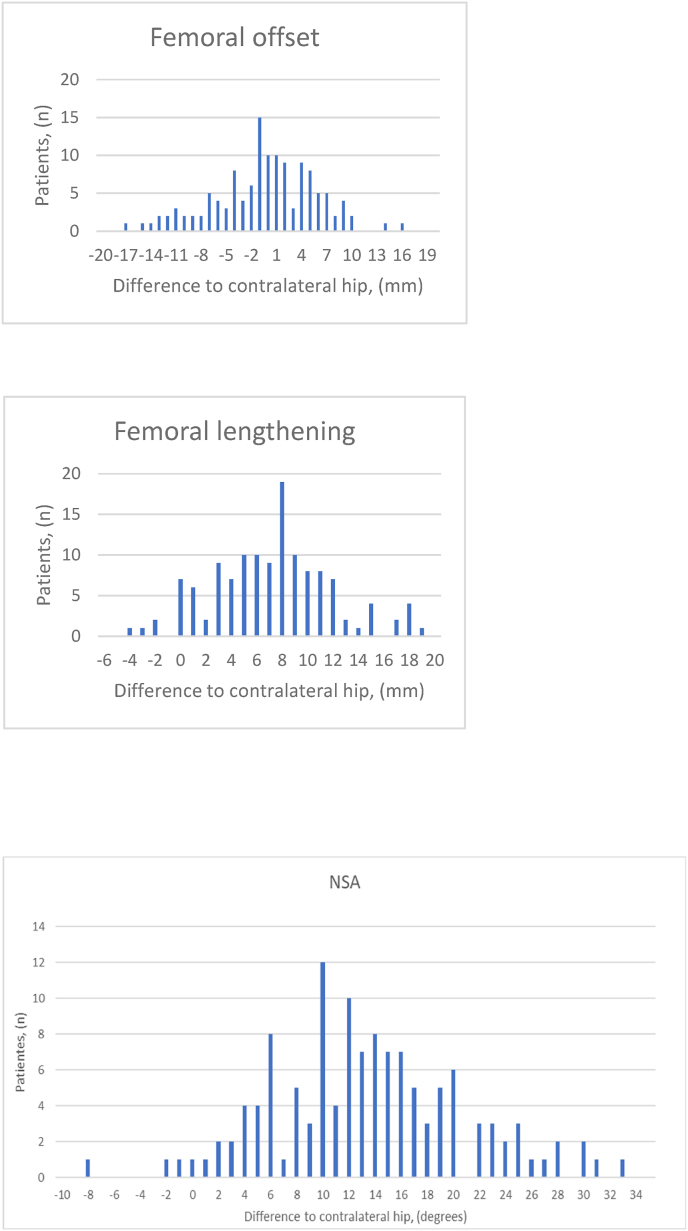
Biomechanical effect at the femoral bone on clinical x-rays.

A mean 0.8 mm decrease in FO (p = 0.34) showed no statistical difference between the operated side and the contralateral side. However, we found a mean 6.8 mm increase in FL (p < 0.001) (See Fig. 5).

**Fig. 6:**
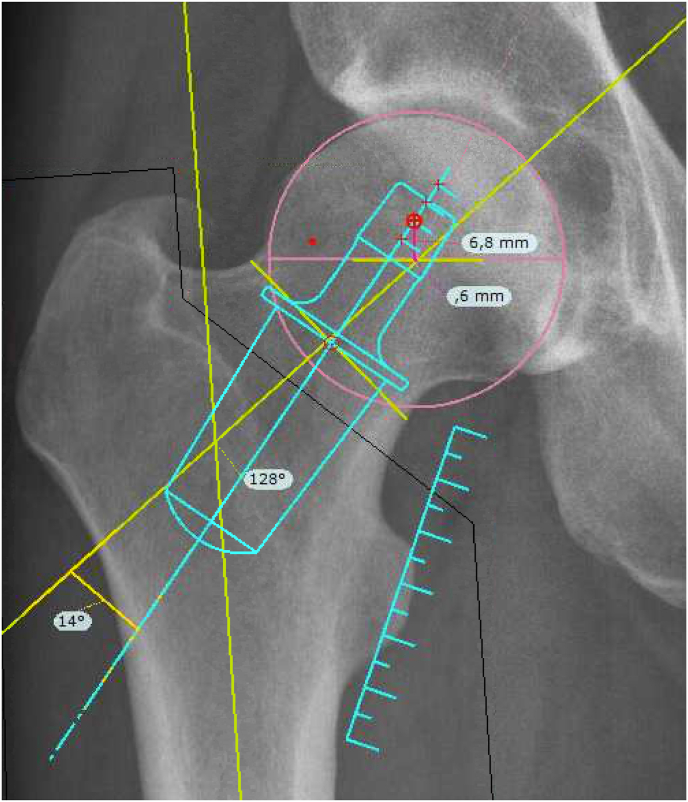
Illustration showing how the mean stem is positioned in the femur.

**Fig. 7:**
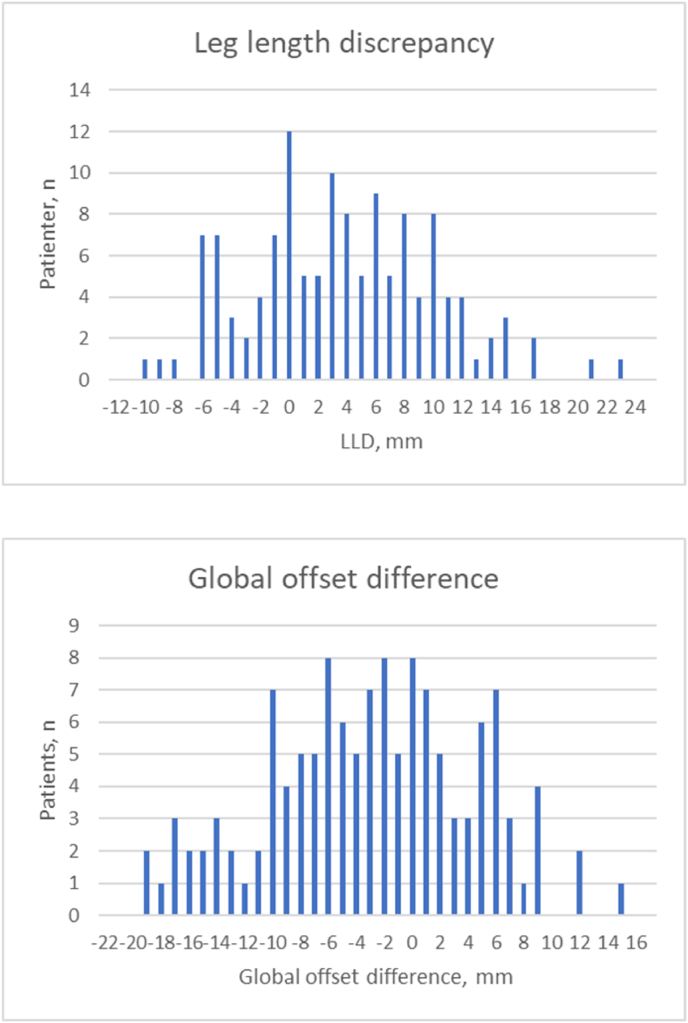
Biomechanical effect on total hip from clinical x-rays.

Leg length recorded a mean 3.8 mm increase (p < 0.001) as a direct measure on pelvic x-ray. We found a mean 14° increase (p < 0.001) in NSA. Lastly, the GO was decreased −3.4 mm (p = 0.002), where the GO was calculated as the FO added to the horizontal pelvic centre of rotation (HCR) (See Fig. 6, Fig. 7).

The intra-observer variation demonstrated excellent reliability; correlation of FO 0.93, GO 0.96, LLD 0.94 and NSA 0.95. The inter-observer variation also demonstrated good to excellent reliability; correlation of FO 0.92, GO 0.86, LLD 0.86 and NSA 0.92 (Table 2).

## 4 Discussion

The overall aim of this study was to evaluate the radiological restoration of hip biomechanics after using a collum-fixated stem in THA, using the contralateral native hip as a reference.

Before the Primoris prosthesis was adopted for clinical use, it was inserted into nine cadaveric femurs, using bone compacting technique. The surgeon hereby gained practical knowledge of the implant and the instruments. We compared the x-rays made before and after surgery to establish how the prosthesis would affect selected femoral hip parameters. Cadaveric FO was +3.4 mm; FL +6.8 mm, thereby counteracting the expected slight medialisation and proximal positioning of the cup. Hereafter, the Primoris stem was introduced into clinical use in a stepwise manner.

On the clinical x-rays, we found a mean 0.8 mm decrease in FO, which showed no statistical difference from the offset registered on the contralateral side. We found no other studies in which a neck-stabilised and neck only classified 2C stem, JISRF, described the effect on FO. However, by broadening the scope of our comparison, we found that the Metha short stem (Braun Aesculap), JISRF 3 A, had a mean 3.6 mm increase^14^; the Nanos short-stem (Smith and Nephew), JISRF 2 A, had a mean 0.6 mm increase in FO^15^ and the Proxima short stem (DePuy), JISRF 2 B, had a mean 0.6, decrease.^18^ An increase in FO is associated with an improved movement range, enhanced functional outcome, better stability and less wear.^7^^,^^8^^,^^19^ To compensate for the slight medialisation of the acetabular COR, an increased FO is preferred. Thereby, a reduction in GO is avoided. A reduction exceeding 5 mm in GO after THA was found to have a negative association with abductor muscle strength.^19^ In the present study, 41% (52/127) of the GO was less than −5 mm, which illustrates that recreating the FO may be one of the challenges associated with a neck-stabilised, neck-only stem.

A mean 6.8 mm increase in femoral length contributed to a mean 3.8 mm leg length increase. This is comparable to other studies on short-stem designs in which a mean increase in leg length was found to be 3.3 mm,^14^ 0.36 mm (15) and 3.1 mm.^18^

Compared with conventional femoral components, the neck osteotomy level is of considerable importance when using a short stem. However, many of the short-stem designs employ a stem that is partly anchored in the femoral diaphysis. A stem which is fixated only in the collum relies on collum osteotomy for optimal placement and fixation. As described previously, the stem was intentionally positioned following the collum axis. However, our study revealed that the stem had a tendency to being positioned 14° more valgus than the contralateral side, which affected FO. However, we should bear in mind that if the stem had been placed in a more neutral or even varus manner, the forces affecting the stem would have been higher, potentially increasing the risk of early fracture or stem-loosening.

On the acetabular side, determining the centre of rotation, we found a horizontal mean medialisation of 2.6 mm and a 3.5 mm vertical mean proximalisation, indicating that the cup was implanted slightly more medially and proximally, respectively. Similar results for cup placement have previously been documented in THA using a standard stem and a short-stem design.^14^

### 4.1 Strengths and limitations

In the present study, we described the placement of a short stem in the femur. This approach provided knowledge of what to bear in mind for the future development, design and surgery with short-stem prostheses. We did not compare the patient-related outcome score with the placement of the prosthesis because of many hip parameter variables were employed in a relatively small group of patients.

A limitation is that this study was conducted retrospectively. Furthermore, all operations were conducted by only two surgeons on a highly selected group. Therefore, the outcomes are difficult to extrapolate.

Another limitation of this study was that two-dimensional radiographs were employed to measure three-dimensional distances, including FO. Even so, radiography is the method of choice as the majority of surgeons use it for preoperative planning and postoperative positioning assessment. Furthermore, radiography is as reliable and accurate as computed tomography for this use.^20^ The ante- or retroversion of the stem was not evaluated due to lack of reproducibility in lateral radiograph measurements.

We compared the operated hip to the contralateral native hip, assuming that the contralateral hip is an inverted image of how the operated hip used to look before arthritic changes influenced the biomechanics.

In THA, a neck-stabilised and neck-only stem enabled hip anatomy restoration to a favourable range with respect to GO and LLD where the mean difference recorded was within ±5 mm. However, FL had a mean difference exceeding 5 mm, and the Primoris stem tended to be implanted in valgus.

## Ethical statement

This retrospective cohort study was conducted at the Farsoe Orthopaedic Clinic, Aalborg University Hospital, Aalborg, Denmark. Our local ethics committee approved the study (approval no. N-20100054) and the study was registered with ClinicalTrials.gov (NCT01326832).

## Funding statement

The authors received no financial support for the research, authorship, and/or publication of this article.

## Patient's consent

All patients provided informed consent and were enrolled according to guidelines for observational studies in epidemiology (Strengthening the Reporting of Observational Studies in Epidemiology (STROBE) and in conformity with the Helsinki Declaration.

## Declaration of competing interest

The authors declare that they have no known competing financial interests or personal relationships that could have appeared to influence the work reported in this paper.
